# The effect of body mass index on short-term outcomes in patients undergoing off-pump coronary artery bypass grafting surgery: a retrospective study from a single cardiovascular center

**DOI:** 10.1186/s13019-024-02586-1

**Published:** 2024-02-11

**Authors:** Chen-ying Ding, Wen-hui Qi, Yu-jie An, Xin Yuan, Yun-tai Yao, the Evidence in Cardiovascular Anesthesia (EICA) Group

**Affiliations:** 1 Department of Anesthesiology, Hohhot First Hospital, Hohhot, 010030 Inner Mongolia Autonomous Region China; 2 Department of Anesthesiology, Harrison International Peace Hospital, Hengshui, 053000 Hebei Province China; 3 Department of Anesthesiology, The Friendship Hospital of Ili Kazakh Autonomous Prefecture, Yining, 835000 Xinjiang Uygur Autonomous Region China; 4 Department of Anesthesiology, Fuwai Hospital, National Center for Cardiovascular Diseases, Peking Union Medical College and Chinese Academy of Medical Sciences, No. 167, Beilishi Road, Xicheng District, Beijing, 100037 China

**Keywords:** Body mass index, Short-term outcomes, Off-pump, Coronary artery bypass grafting

## Abstract

**Objective:**

This study is designed to investigate the impact of body mass index (BMI) on the short-term outcomes of patients undergoing off-pump coronary artery bypass graft (OPCAB) surgery.

**Methods:**

Data was obtained from 1006 Chinese patients who underwent isolated, primary OPCAB at a high-traffic cardiovascular center during 2020. Subjects were categorized, by BMI, into a low & normal weight (LN) group (BMI < 24 kg/m^2^), an overweight (OVW) group (24 ≤ BMI < 28 kg/m^2^), and an obese (OBS) group (BMI ≥ 28 kg/m^2^). Information pertaining to patients’ short-term outcomes (including incidence of mortality and morbidities; duration of postoperative mechanical ventilation; length of stay in the ICU and hospital; postoperative bleeding; etc.) were extracted, and the data from each group were compared.

**Results:**

The incidences of in-hospital mortality and morbidities were similar for all three groups. The volume of fluid infusion, postoperative bleeding within 24 h and total bleeding in LN group were higher than those in the OBS group (*P* < 0.001). The hemoglobin level was lower in the LN group than that in the OBS group (*P* < 0.001). Duration of mechanical ventilation and length of stay in the ICU in the LN group were longer than those in the OBS group (*P* < 0.001).

**Conclusions:**

Our results demonstrate that BMI is not significantly related with short-term outcomes in OPCAB patients. However, we suggest that OPCAB patients with low-normal BMI are more susceptible to post-operative blood loss.

## Introduction

Obesity, one of the most prevalent public health problems globally, is a direct cause of cardiovascular risk factors (*e.g.,* dyslipidemia, type 2 diabetes, hypertension, and sleep disturbances). This century has seen a dramatic increase in incidence of obesity in both developed countries and developing countries, coupled with the average onset age steadily decreasing [1, 2]. For example, in China from 1993 to 2015, the prevalence of obesity in children and adolescents increased from 5.0 to 19.3% [3]. The relationship between obesity and life-expectancy is complex, with the consensus of studies showing increased mortality in people who are either underweight or very obese [4, 5]. BMI is calculated by dividing a person's body weight (kg) by the square of their height (m^2^) and is a commonly used obesity indicator. Studies have shown that obese patients undergoing coronary artery bypass grafting (CABG) are not at higher risk of perioperative death or other adverse outcomes compared to patients with body mass within the normal range [6]. However, multiple studies have shown that underweight patients are at greater risk of death and complications after CABG surgery [7, 8]. Furthermore, elevated BMI has been shown to be a protective factor in the reduction in mortality at 30 days after CABG [9]. The results of a multivariate regression analysis showed that obesity was significantly associated with risk of only superficial sternum wound infection, leg infection and atrial arrhythmias [10].

It is unknown whether BMI is an isolated risk factor for short-outcomes of off-pump coronary artery bypass grafting (OPCAB). The current study is designed to investigate the effects of BMI on in-hospital outcomes of Chinese patients undergoing isolated, primary OPCAB.

## Methods

### Ethical approval

The study was approved by the Ethical Committee of Fuwai Hospital (2019-1301). Given the retrospective nature of the study, patient consent was waived.

### Study design and patient population

Patients who received primary and isolated OPCAB at Fuwai Hospital during 2020 were retrospectively included.

The inclusion criteria were: (1) age: > 18 years old; (2) procedure: primary and isolated OPCAB. The exclusion criteria were: (1) emergency OPCAB; (2) OPCAB performed in conjunction with other surgeries; (3) patients whose outcomes were missing or incomplete; (4) prior cardiac surgery. All included patients were not asked to lose weight during hospitalization.

Included patients were divided into three groups according to BMI. Studies have shown that Chinese have lower BMI than Europeans because Chinese have a lower metabolism associated with obesity [11].

Therefore, we used the Working Group on Obesity in China (WGOC) and the Guidelines for the Prevention and Control of Overweight and Obesity in Chinese Adults [12] to define overweight or obesity, as follows: BMI < 18.5 kg/m^2^ (Underweight), 18.5 ≤ BMI < 24 kg/m^2^ (Normal), 24 ≤ BMI < 28 kg/m^2^ (Overweight) and BMI ≥ 28 kg/m^2^ (Obese).

In this study, only 7 patients were in the underweight group, thus we combined the underweight group and normal weight group to create the “low and normal” group (< 24 kg/m^2^). Hence the final groupings are BMI < 24 kg/m^2^ (low & normal weight group), 24 ≤ BMI < 28 kg/m^2^ (overweight), and BMI ≥ 28 kg/m^2^ (obese).

### Operative techniques

All surgical and anesthesia procedures followed standard protocols: (1) patients take antihypertensive and antianginal drugs until the morning of surgery and (2) antiplatelet and anticoagulant drugs are discontinued on admission and replaced with low molecular weight heparin which is continued until the day before surgery. As per standard institutional requirements, all surgeons must be specialized in congenital or valve heart surgery for > 3 years before undertaking any CABG procedures. Fuwai Hospital has always been at the forefront of this type of surgery, pioneering the first OPCAB to be performed in China in 1996. The hospital maintains its pre-eminence by requiring surgeons to perform at least 100 CABG procedures before they are considered qualified to perform OPCAB. Once qualified, the choice of OPCAB, as opposed to CABG, for a particular patient is usually at the discretion of the individual surgeon.

Standardized anesthesia techniques include sufentanil (1.5–2.0 μg/kg), midazolam (0.1 mg/kg), rocuronium bromide (0.6–1 mg/kg), and sevoflurane (0.5–2.5%). In this study, all patients had undergone similar procedures including median sternotomy, followed by wide opening of the right pleural space, and pericardial notching at the level of the diaphragm well above the phrenic nerve; the right diaphragmatic fat pad is removed in all patients to further enable rotation. The target vessels are inspected to determine the number of grafts to be performed, and to determine if the procedure can be indeed completed off-pump; left internal mammary artery tissue samples being routinely collected; collection of large saphenous bundle by standard open methods when required; and a 200 IU/kg dose of heparin administered to obtain an activated clotting time > 300 s. Finally, all patients underwent intraoperative cell salvage with autotransfusion of washed, salvaged red blood cells at the end of the operation.

### Data collection

Data were collected from hospital electronic medical records and included laboratory test results, perioperative condition, and postoperative recovery. After surgery, the patient was transferred to ICU, where the ICU nurse recorded the duration of mechanical ventilation, chest tube drainage, and other conditions of the patient.

### Outcome events definition

The primary outcome was the composite incidence of in-hospital mortality and morbidities. For morbidities, we referenced the definitions for cardiovascular endpoint events formulated by the American Heart Association [13, 14].

*Non-fatal myocardial infarction* is defined as new or presumed new significant ST-segment-T wave (ST-T) changes or new LBBB on the ECG or development of pathological Q waves on the ECG.

*Low cardiac output* is defined as cardiac index (CI) < 2.0 L/min/m^2^; systolic blood pressure < 90 mmHg; tissue hypoperfusion without hypovolemia; left ventricular assist (LVAD), intra-aortic balloon pump (IABP) and inotropic support after surgeries.

*Non-fatal stroke* is defined as central neurological deficit persisting > 72 h.

*Continuous renal replacement therapy* is defined as a patient having acute kidney injury (AKI) or acute renal failure (ARF) after surgery requiring dialysis treatment.

*Pneumonia* is defined as a positive result of bronchial lavage fluid or sputum culture, or changes in chest X-ray.

We used chest tube drainage in the first 24 h after admission to ICU as an indicator of postoperative bleeding. Chest tube drainage was divided by body weight to obtain chest tube drainage/weight.

### Statistical analysis

All descriptive data are presented as either mean (standard deviation [SD]) for normally distributed continuous variables, median (interquartile range) for abnormally distributed continuous data, or number (percent) for categorical data, as appropriate. Quantitative data were analyzed using one-way ANOVA if meeting the normal distribution, and Fisher’s least significant difference (LSD) mehtod was applied for post hoc comparisons. The Wilcoxon Rank Sum test was used for quantitative data that did not satisfy the normal distribution. Comparisons of categorical variables were performed using Chi-square tests.

All statistical analyses were performed with SPSS version 23.0 software (SPSS Inc., Chicago, IL, USA). A two-sided value of *P* < 0.05 was considered to indicate statistical significance.

## Results

### Baseline characteristics

**Fig. 1:**
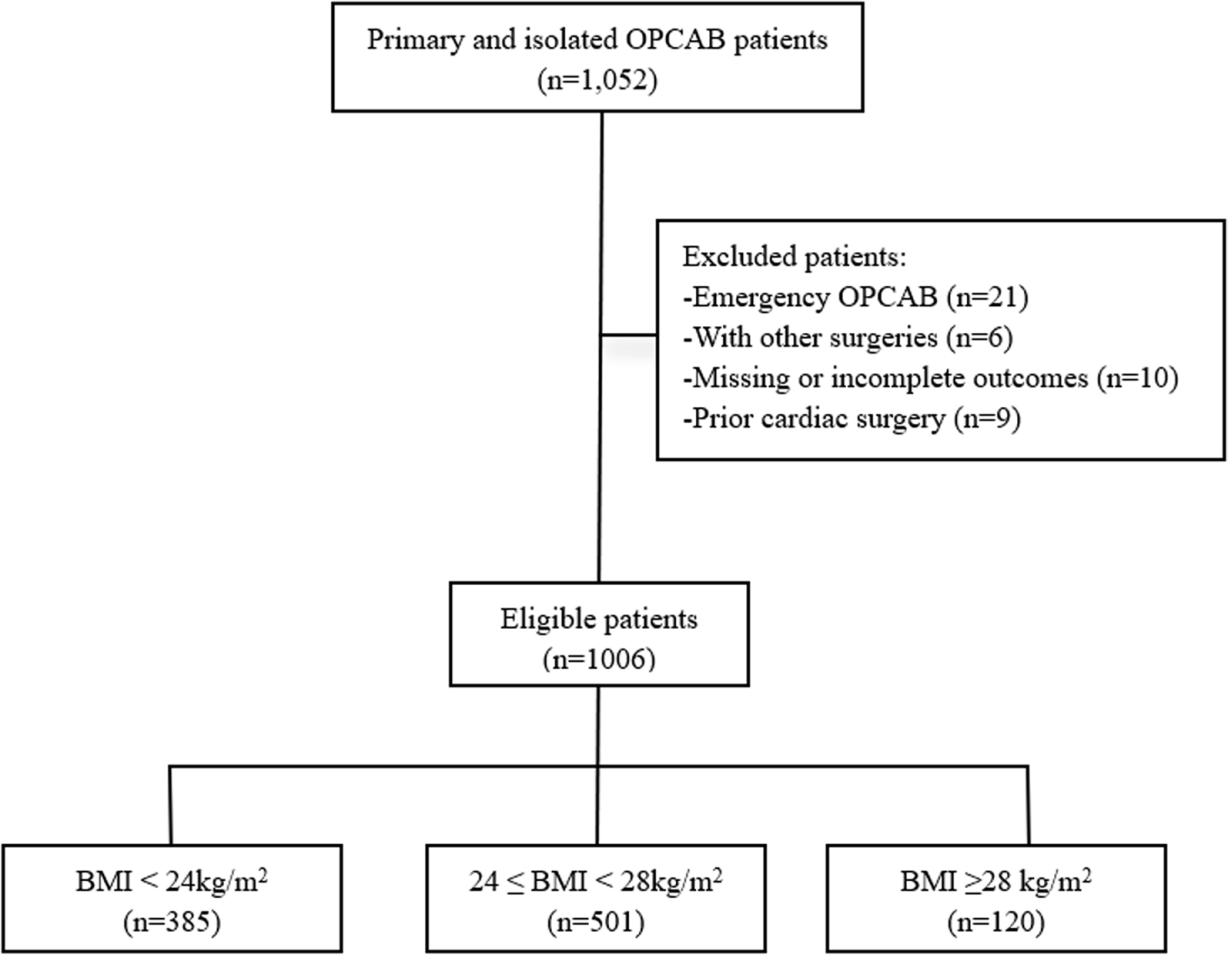
Enrollment flowchart

**Fig. 2:**
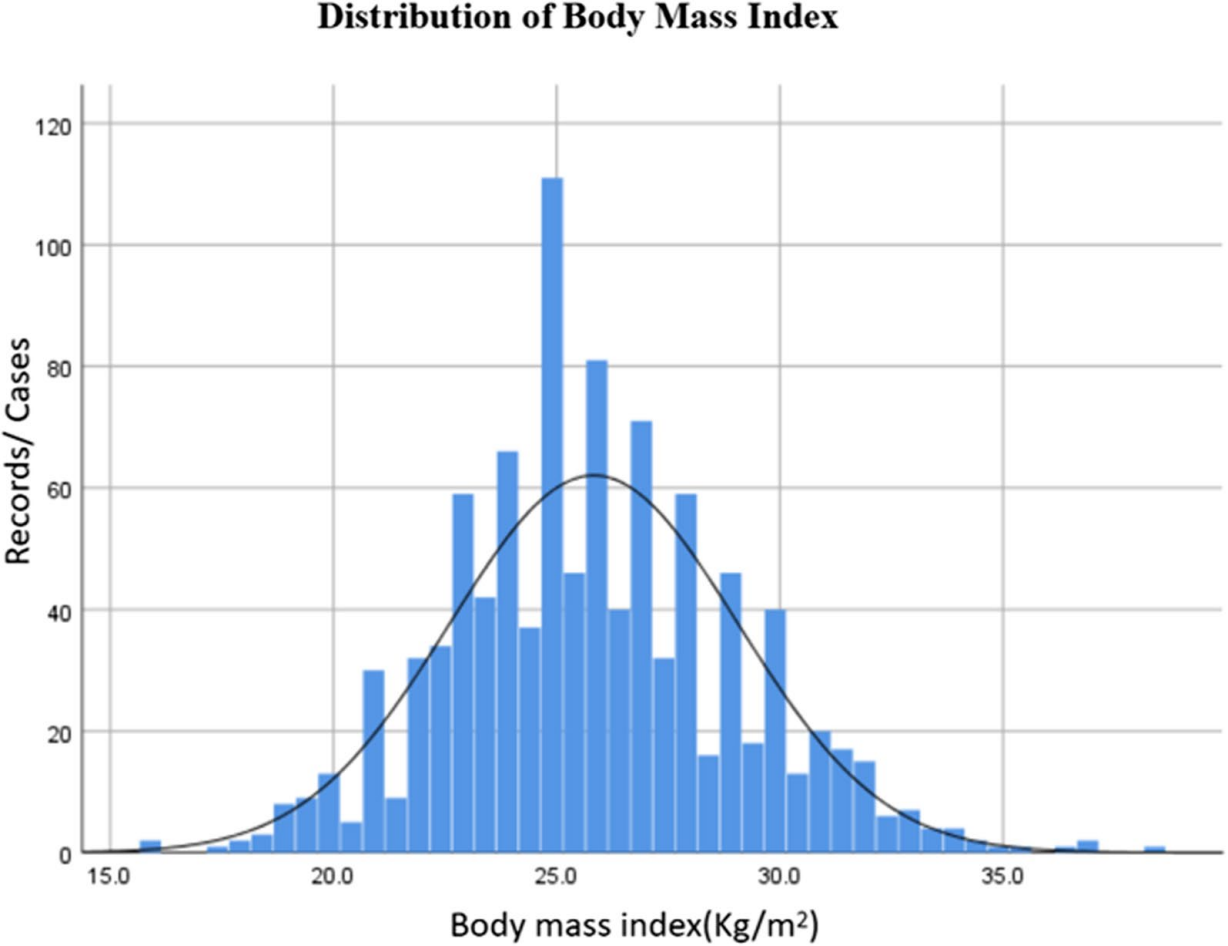
BMI distribution across the analyzed sample shows an approximately normal distribution as seen by the curve

The patient selection process is shown in Fig. 1. BMI of the included patients shows an approximately normal distribution (Fig. 2). The patients of under-normal weight tended to be older compared with the overweight and obese groups (63.8 ± 8.2 vs. 61.2 ± 9.1, 59.3 ± 9.7, *P* < 0.001). The underweight group also had the highest proportion of female patients (31.8% *vs.* 18.3%, 20.1%, *P* < 0.001) (Table 1). Among the included risk factors, smoking and drinking are more common in overweight and obese patients (42.2% vs. 54.8%, 51.0%; 40.1% vs. 51.2%, 48.6%) However, preoperative myocardial infarction incidence was lower in low-normal weight group. (29.2% *vs.* 40.2%, 38.6%,* P* = 0.009).

**Table 1:** Baseline characteristics

Variables	Group LN (n = 385)	Group OVW (n = 501)	Group OBS (n = 120)	*P*	*P*1	*P*2	*P*3
Age	63.8 (8.2)	61.2 (9.1)	59.3 (9.7)	< 0.001*	< 0.001*	< 0.001*	0.008*
Male	189 (68.2)	392 (81.7)	199 (79.9)	< 0.001*	< 0.001*	0.003*	0.618
Risk factors							
Smoking	117 (42.2)	263 (54.8)	127 (51.0)	0.004*	0.001*	0.054	0.348
Drinking	111 (40.1)	246 (51.2)	121 (48.6)	0.011*	0.002*	0.052	0.532
Chronic obstructive pulmonary disease	4 (1.4)	6 (1.3)	1 (0.4)	0.532	-	-	-
Hypertension	185 (66.8)	311 (64.8)	180 (72.3)	0.121	-	-	-
Diabetes mellitus	110 (39.7)	197 (41.0)	92 (36.9)	0.565	-	-	-
Dyslipidemia	230 (83.0)	392 (81.7)	215 (86.3)	0.284	-	-	-
Atrial fibrillation	11 (4.0)	14 (2.9)	8 (3.2)	0.784	-	-	-
Myocardial infarction	81 (29.2)	193 (40.2)	96 (38.6)	0.009*	0.002*	0.027*	0.09
Percutaneous coronary intervention	23(8.3)	44(9.2)	17 (6.8)	0.553	-	-	-
Pulmonary hypertension	3(1.1)	7 (1.5)	3 (1.2)	0.937	-	-	-
Peripheral vascular diseases	39 (14.1)	79 (16.5)	36 (14.5)	0.628	-	-	-
Neurological events	30 (10.8)	37 (7.7)	13 (5.2)	0.056	-	-	-
LVEF	59.0 (7.6)	58.8 (6.9)	59.6 (7.0)	0.364	-	-	-
Creatinine (mmol/L)	85.0 (22.7)	87.6 (19.6)	87.3 (20.6)	0.239	-	-	-

Values are median (SD) or n (%). LVEF = Left ventricular ejection fraction. *P*1, Group LN versus Group OVW; *P*2, Group LN versus Group OBS; *P*3, Group OVW versus Group OBS;

* means a difference on comparison with two groups with *p* < 0.05

### In-hospital mortality and morbidities

There was no difference in adverse events characteristics (Table 2). Significantly, mortality and morbidities were also similar among groups. In addition, cardiac arrest, new pacemaker, atrial fibrillation, non-fatal myocardial infarction, low cardiac output, IABP (Intra-aortic balloon pump), ECMO (Extracorporeal membrane oxygenation), non-fatal stroke, CRRT (Continuous renal replacement therapy), and pneumonia showed similar occurrence across the three cohorts. Overall mortality was 0.001% (1/1006) in the analyzed sample, the only death occurring in the LN group (Table 2).

**Table 2:** Adverse events characteristics

Variables	Group LN (n = 385)	Group OVW (n = 501)	Group OBS (n = 120)	*P*
Mortality and morbidities	6 (2.2)	12 (2.5)	8 (3.2)	0.735
Mortality	1 (0.4)	0 (0.0)	0 (0.0)	0.523
Any morbidity	5 (1.8)	12 (2.5)	8 (3.2)	0.588
Cardiac arrest	1 (0.4)	2 (0.4)	0 (0.0)	0.804
Atrial fibrillation	1 (0.4)	4 (0.8)	4 (1.6)	0.338
New pacemaker	3 (1.1)	2 (0.4)	2 (0.8)	0.486
Non-fatal myocardial infarction	0 (0.0)	2 (0.4)	1 (0.4)	0.616
Low cardiac output	4 (1.4)	7 (1.5)	3 (1.2)	1
Intra-aortic balloon pump	1 (0.4)	4 (0.8)	0 (0.0)	0.525
Extracorporeal membrane oxygenation	4 (1.4)	10 (2.1)	3 (1.2)	0.700
Non-fatal stroke	0 (0.0)	2 (0.4)	3 (1.2)	0.120
Continuous renal replacement therapy	7 (2.5)	15 (3.1)	6 (2.4)	0.803
Pneumonia	151 (54.5)	265 (55.2)	129 (51.8)	0.683

Values are n (%)

### Intraoperative and postoperative characteristics

The infused fluid, postoperative bleeding within 24 h and total bleeding in the LN group were markedly higher compared with the OBS group (Table 3, *P* < 0.001). After surgery, hemoglobin levels were lower in the LN group than in the OVW and OBS groups (114.7 ± 57.7 vs. 116.2 ± 17.6, 118.4 ± 18.4). Compared with the OVW and OBS group, duration of mechanical ventilation and length of stay in the ICU in the LN group was longer (20.6 ± 15.5 vs. 17.5 ± 11.4, 18.4 ± 12.8, *P* < 0.001) and (70.2 ± 47.0 vs. 66.7 ± 58.9, 64 ± 44.0, *P* < 0.001), respectively. Intraoperative, utilization of cardiovascular medications as well as duration of surgery and anesthesia were not significantly different between the three groups. After adjusting for other independent risk factors of mortality in the multivariate analysis, BMI as a continuous variable was not an independent predictor of mortality (Table 4).

**Table 3:** Operative and postoperative characteristics

Variables	Group LN (n = 385)	Group OVW (n = 501)	Group OBS (n = 120)	*P*	*P*1	*P*2	*P*3
Operative characteristics							
Fluid input (ml/kg)	24.5 (10.8)	19.9 (9.2)	21.6 (7.1)	< 0.001*	< 0.001*	< 0.001*	0.027*
Epinephrine	29 (10.5)	55 (11.5)	24 (9.6)	0.741	-	-	-
Dopamine	144 (52.0)	240 (50.0)	141 (56.6)	0.238	-	-	-
Milrinone	24 (8.7)	32 (6.7)	17 (6.8)	0.580	-	-	-
Norepinephrine	54 (19.5)	86 (17.9)	44 (17.7)	0.839	-	-	-
Nitroglycerin	178 (64.3)	318 (66.3)	168 (67.5)	0.735	-	-	-
Surgery duration, min	208.6 (47.0)	206.9 (46.7)	213.5 (45.1)	0.188	-	-	-
Anesthesia duration, min	259.3 (51.8)	255.1 (48.8)	263.7 (50.1)	0.083	-	-	-
Postoperative characteristics							
Bleeding within 24 h (ml/kg)	9.0 (5.2)	8.0 (6.1)	6.9 (4.8)	< 0.001*	0.022*	< 0.001*	0.011*
Total bleeding (ml/kg)	17.9 (11.7)	14.9 (9.9)	12.1 (8.2)	< 0.001*	< 0.001*	< 0.001*	0.001*
Chest drainage duration, d	4.7 (2.2)	4.7 (2.4)	4.6 (1.8)	0.767	-	-	-
Reoperation for bleeding	3 (1.1)	6 (1.3)	2 (0.8)	0.927	-	-	-
Postoperative hemoglobin (g/L)	114.7 (57.7)	116.2 (17.6)	118.4 (18.4)	< 0.001*	0.001*	< 0.001*	0.353
Postoperative platelet count (10^9^/L)	187.7 (63.8)	187.3 (55.6)	192.9 (61.7)	0.451	-	-	-
Red blood cell transfusion rate	9 (3.2)	5 (1.0)	5 (2.0)	0.095	-	-	-
Fresh frozen plasma transfusion rate	10 (3.6)	9 (1.9)	6 (2.4)	0.341	-	-	-
Platelet concentrates transfusion rate	0	0	0	NA	-	-	-
Any transfusion rate	16 (5.8)	14 (2.9)	9 (3.6)	0.145	-	-	-
Mechanical ventilation duration (h)	20.6 (15.5)	17.5 (11.4)	18.4 (12.8)	0.006*	0.001*	0.001*	1
Length of stay in ICU (h)	70.2 (47.0)	66.7 (58.9)	64.5 (44.2)	< 0.001*	0.001*	0.001*	0.056
Readmission to ICU	5.0 (1.8)	4 (0.8)	3 (1.2)	0.891	-	-	-
Length of stay in the hospital, d	8.3 (2.9)	8.5 (5.4)	8.54 (4.3)	0.832	-	-	-

Values are median (SD) or n (%). P1, Group LN versus Group OVW; P2, Group LN versus Group OBS; P3, Group OVW versus Group OBS

* means a difference on comparison with two groups with *p* < 0.05

**Table 4:** Multiple logistic regression for complications

Predictor variable	OR (95% CI)	*P*
Male	2.427 (0.650–9.065)	0.187
Age	0.996 (0.959–1.035)	0.847
Body mass index	0.922 (0.686–1.239)	0.590
Smoking	1.952 (0.222–17.160)	0.546
Drinking	1.417 (0.170–11.787)	0.747
Hypertension	1.229 (0.496–3.049)	0.656
Diabetes	1.327 (0.562–3.130)	0.519
Dyslipidemia	1.010 (0.340–3.001)	0.985
Myocardial infarction	0.440 (0.136–1.424)	0.170
Left ventricular ejection fraction	1.037 (0.975–1.102)	0.245
Length of stay in ICU	1.007 (0.997–1.018)	0.147
Mechanical ventilation duration	0.942 (0.872–1.018)	0.130
Postoperative hemoglobin	1.004 (0.997–1.011)	0.277
Postoperative platelet count	0.997 (0.990–1.004)	0.444
Postoperative bleeding within 24 h/weight	0.998 (0.969–1.028)	0.889
Intraoperative total fluid	0.992(0.976–1.008)	0.315

## Discussion

It is well established that obesity is one of the most pressing public health problems in the world. Obesity is inextricably linked to hypertension, diabetes, and cardiovascular disease, leading to an increasing number of obese patients undergoing heart surgery. This demographic accounted for 11.9% of patients in our study. Although obesity is a predisposing factor for many chronic diseases, multiple studies [15, 16] have shown that excessive obesity may improve outcomes for patients with coronary heart disease (known as the "obesity paradox"). In particular, Le-Bert et al*.* [17] demonstrated that this paradox was present among elderly obese patients undergoing CABG by median sternotomy.

Similar studies [18] have shown that obese patients are (i) more likely to be found in younger, male populations and (ii) more likely to smoke or drink. However, in our study, coronary heart disease risk factors such as hypertension, diabetes, hyperlipidemia, myocardial infarction were similar in all three groups, which may be caused by the small sample size of the obese group. These findings suggest that obese patients tend to develop coronary artery disease earlier and are more prone to myocardial infarction, as well as surgical revascularization, at a younger age.

The current study shows that BMI is not a contributing factor in mortality and/or complications in patients undergoing OPCAB surgery. This is similar to the findings of Bhamidipati et al*.* [18] who showed that elevated BMI (≥ 30 kg/m^2^) is not an independent influence on complications and mortality among OPCAB patients. However, a nationwide study with a systematic review and meta-analysis shows a U-shaped association between mortality and body mass index classes, with lower mortality in overweight and obese class I and II patients relative to normal weight patients and increased mortality in underweight and obese class III individuals [19].

Meanwhile, we found that there was no significant difference in the length of stay in the hospital among the three groups following OPCAB. Interestingly, compared with the obese group, the low-normal weight group had more intraoperative fluid infusion and postoperative bleeding and required more blood products, which is consistent with previous reports [20, 21]. In addition, the length of stay in the ICU was also significantly prolonged for patients in the low-normal weight group. Recently, it has been demonstrated that adipocytes are able to produce plasminogen activator inhibitor-1 (plasminogen is inactive until converted to plasmin and breaks down fibrin clots), possibly explaining why obese people have less perioperative bleeding [22]. In addition, less hemodilution in obese patients may also contribute to lower risk of postoperative bleeding. However, one study has demonstrated that obesity was not associated with lower risk of bleeding and the procoagulant hemostatic profile in obese individuals may not be sufficient to protect against clinically relevant bleeding [23, 24]. Nevertheless, BMI is widely accepted as an indicator to predict postoperative blood loss and transfusion.

Studies by Ghanta et al. [25] have shown that morbidly obese patients (BMI > 40 kg/m^2^) have a mortality rate nearly 60% higher than patients falling within the normal weight range and have > twofold increase in renal failure and > 6.5-fold increase in infection of deep sternal wounds. Due to the significant difference in body fat percentage of Chinese patients compared with their European and American counterparts, we have very few morbidly obese patients. Therefore, no adverse events related to excess weight were observed in this study.

Previous studies have shown that underweight patients exhibit a higher incidence of postoperative renal insufficiency, longer duration of mechanical ventilation, and longer ICU stay [9, 26, 27]. Zittermann et al*.* [26] found that compared with normal and overweight patients, the multivariable-adjusted hazard ratio of 2-year mortality was higher in underweight patients. In our study population, there were only 7 patients classified as underweight, thus they were combined with patients with BMI within normal range, making statistical inference problematic. In our study, the length of stay in the ICU and duration of mechanical ventilation were significantly longer for patients in the low-normal group than in the overweight groups.

## Limitations

Firstly, this is a retrospective study with the limitations inherent in retrospective studies. Secondly, we used a BMI-based definition of obesity in China, which has a lower cut-off value than the Western population, and our results are suitable for Chinese. Thirdly, because this is a single-center study, our sample size is relatively small and may not be sufficient to highlight certain statistical differences.

## Conclusions

The results of this study suggest that obesity is not associated with increased risk of postoperative mortality and complications in Chinese patients undergoing primary and isolated OPCAB. However, lower body weight might be indicative of an increased risk of both incidence and profuseness of intraoperative bleeding for these patients. More extensive studies are warranted to validate the above results.

## Data Availability

The original contributions presented in the study are included in the article, further inquire can be directed to the corresponding author.
